# Flexible Composites Based on PEDOT:PSS for Environmentally Friendly Electrocardiography Electrodes

**DOI:** 10.3390/polym18080947

**Published:** 2026-04-12

**Authors:** María Elena Sánchez Vergara, José Miguel Rocha Flores, Marisol Martinez-Alanis, Selma Flor Guerra Hernández, Ismael Cosme

**Affiliations:** 1 Faculty of Engineering, Universidad Anahuac México, Av. Universidad Anáhuac 46, Col. Lomas Anáhuac, Huixquilucan 52786, Mexico; jose_rocha@anahuac.mx (J.M.R.F.); marisol.martinez2@anahuac.mx (M.M.-A.); 2 Fidel Velázquez Technological University, Emiliano Zapata S/N, Col. El Trafico, Nicolás Romero 54400, Mexico; 3 National Institute of Astrophysics, Optics and Electronics (INAOE), Luis Enrique Erro # 1, Tonantzintla 72840, Mexico; selmaf@inaoep.mx (S.F.G.H.); ismaelcb@inaoep.mx (I.C.)

**Keywords:** PEDOT:PSS composite, cellulosic substrates, mechanical properties, optical properties, wearable electrocardiogram

## Abstract

Wearable electrodes have attracted attention for their ability to monitor human electrophysiological signals, such as those generated by the heart and captured via electrocardiography (ECG). In this study, an easy and scalable drop-coating method was used to develop flexible, dry, and sustainable ECG electrodes composed of a poly(3,4-ethylenedioxythiophene):poly(4-styrenesulfonate) (PEDOT:PSS)/polyvinyl alcohol/xanthan gum (**PXP**) composite. The electrodes were fabricated on different cellulosic substrates, such as Xuan paper, Kraft paper, and wheat bagasse, and further modified through the incorporation of MoO_3_ (**PXPM** composite). **PXP** exhibits a broad absorption band of 350–550 nm, while **PXPM** shows a shifted band of 400–750 nm, due to the interaction of MoO_3_ with PEDOT:PSS. The fluorescence emission of **PXP** appears at 443 nm, while the emission for **PXPM** is broader and centered at 437 nm. Electrically, both composites exhibit continuity and ohmic behavior. Microstructural analysis revealed that the interaction between the composite film and the substrate strongly influences pore formation, film uniformity, and the distribution of Mo species, highlighting the role of MoO_3_ as an interfacial modifier that promotes smoother and more homogeneous coatings on selected cellulosic substrates. All fabricated electrodes demonstrated the capability to detect ECG signals with sufficient quality to be clinically valid.

## 1. Introduction

Due to the rising prevalence of cardiovascular diseases, innovative devices are being developed to help monitor cardiac activity during the different activities a person performs throughout the day [1]. To this end, traditional electrocardiography (ECG) systems are used to capture biopotential signals by placing electrodes on the skin [1,2]. These electrical signals are then processed using a digital filtering system, enabling the analysis of the heart’s behavior through the resulting ECG waveforms [3]. For instance, in the case of a standard twelve-lead ECG used for diagnostic purposes, six electrodes are placed on the chest and four more on the extremities, for a total of ten physical electrodes. The term “twelve-lead” refers to the different electrical vectors, or views, of the heart derived from the mathematical combinations of the signals captured by these ten electrodes [1,2,3].

In most traditional ECG systems, silver/silver chloride (Ag/AgCl) electrodes are placed on the skin to capture the corresponding signals [1,2]. To optimize conductivity and reduce skin–electrode impedance, these electrodes are often accompanied by a conductive gel and an adhesive layer. However, prolonged use can cause adverse effects on the skin, such as irritation, allergies, or discomfort [1,2]. Furthermore, the conductive gel tends to dry out, leading to increased contact impedance and decreased signal quality, which limits its application in long-term continuous monitoring processes [1,2]. Reusable suction-cup electrodes are also used in hospital settings; however, they are often uncomfortable and can cause skin irritation. Continuous disinfection is essential, as improper cleaning can spread resistant pathogens.

To overcome these limitations, dry electrodes have been developed from nanomaterials and conductive polymers, seeking to improve flexibility and adhesion [4]. One example is an innovative, flexible, gel-free electrode formulated with multi-walled carbon nanotubes (MWCNTs) and polydimethylsiloxane (PDMS). Using a solvent-assisted ultrasonic dispersion method, a homogeneous mixture was achieved, reaching 5.5% by weight of MWCNT. The skin–electrode contact impedance was lower than that of traditional Ag/AgCl electrodes when obtaining signals below 100 Hz. Furthermore, the MWCNT/PDMS electrodes demonstrated excellent resistance to motion artifacts during daily monitoring, maintaining signal quality after seven days of use without showing side effects on the skin, such as redness or swelling [5]. Another example is the development of fully organic, self-adhesive, flexible, and highly conductive dry electrodes obtained by solution processing biocompatible blends of poli(3,4-etilendioxitiofeno):poli(4-estirenosulfonato) (PEDOT:PSS), water-based polyurethane (WPU), and D-sorbitol. These electrodes feature high conductivity and skin-adaptive elasticity, offering good adhesion in both dry and wet conditions. The resulting dry electrode exhibits lower skin contact impedance and significantly lower noise levels in both static and dynamic measurements when compared to other dry electrodes described and conventional Ag/AgCl gel electrodes [6]. Similarly, the literature suggests using PEDOT:PSS-treated textile electrodes on fabrics such as cotton, cotton–polyester, Lycra, and polyester versus commercial silver-plated nylon fabric and standard Ag/AgCl disposable electrodes [7,8]. Results indicate that PEDOT:PSS-treated textile electrodes are suitable for recording ECG signals with an error rate of less than 2% and stable performance even after 36 h of continuous use [7].

PEDOT:PSS is a polymer widely employed in electronic devices due to its mixed conduction which results from the presence of two distinct physical phases: the PEDOT phase that, thanks to the *π*-conjugated chains, accounts for electronic transport and the PSS phase that accounts for ionic transport [9,10]. Furthermore, the structural organization of PEDOT:PSS gives it a porous structure that allows for facile doping of the polymer to enhance its conductivity [11]. However, some mechanical limitations have been observed with PEDOT:PSS-based electrodes, such as limited elongation, reduced self-adhesion over time, and instability in aqueous environments [8,12,13]. To address these limitations, composites have been manufactured based on PEDOT:PSS with functional additives, aiming to tailor the polymer’s mechanical, electrical, and adhesion properties [8,12,14,15], even for monitoring during physical activity [15].

This work presents the development of fully organic, flexible, sustainable, and conductive dry electrodes obtained through the solution processing of PEDOT:PSS mixtures, both in their pristine state and modified with MoO_3_. This oxide possesses a wide bandgap and good electrochromic properties; specifically, its thin films turn gray upon detecting an electrical input [16,17,18,19], enabling its use in diverse applications such as catalysis, gas detection devices, organic photovoltaic cells, organic light-emitting diodes, and battery electrodes. Therefore, this study proposes the use of MoO_3_ to enhance the mechanical properties and improve the electrical performance of the fabricated electrodes. Furthermore, unlike previously reported approaches [8,20,21,22,23], these electrodes were deposited onto highly sustainable and unconventional substrates, including wheat bagasse, Kraft paper, and Xuan paper, significantly expanding the possibilities for integration into portable, environmentally friendly devices.

## 2. Materials and Methods

### 2.1. Preparation of Composites

The reagents were obtained from Sigma-Aldrich (St. Louis, MO, USA): poly(3,4-ethylenedioxythiophene):poly(styrenesulfonate) (PEDOT:PSS: [C_8_H_8_O_3_S]-[C_6_H_6_O_2_S]_n_) in 0.8% in H_2_O; poly(vinyl alcohol) (PVA; [−CH_2_−CH(OH)−]_n_), 87–89% hydrolyzed; glycerol (propane-1,2,3-triol; C_3_H_8_O_3_), a hygroscopic solvent with purity ≥ 99.5%; and molybdenum(VI) oxide (MoO_3_, orthorhombic crystal system). Xanthan gum, a polysaccharide with the repeating unit β-D-glucose-(1→4)-β-D-glucose-(1→4)-α-D-mannose-(1→3)-β-D-glucuronic acid-(1→2)-β-D-mannose (empirical formula: [C_35_H_49_O_29_]_n_), was obtained from a commercial source (Verdessence™ Xanthan, Cosmopolita, Mexico City, Mexico).

To prepare the aqueous PVA solution, 0.2 g of PVA powder was dissolved in 9.0 mL of distilled water. The solution was placed in a beaker and stirred with a magnetic mixer at 90 °C for approximately 10 min. Another solution was prepared by adding 0.2 g of xanthan gum powder to 9.0 mL of distilled water and then vigorously stirred at 100 °C for approximately 15 min to obtain a uniform mixture. Subsequently, the aqueous PVA solution was added to the aqueous solution with xanthan gum. The resulting mixture was combined with an aqueous solution containing 4.5 g of glycerol and 9.0 g of PEDOT:PSS. The hybrid solution was vigorously stirred at 80 °C for 40 min to obtain the composite identified as **PXP**. It is important to consider that both xanthan gum and PVA act as thickeners and as glue, while glycerol has the function of a plasticizer.

The second composite was synthesized following the exact same process and using identical amounts of distilled water, PVA, xanthan gum, glycerol and PEDOT:PSS. Immediately after adding the PEDOT:PSS, 0.09 g of MoO_3_ powder suspended in 3.1 mL of distilled water was added. The mixture was then stirred at 80 °C for 40 min, resulting in the composite designated as **PXPM**.

**Figure 1:**
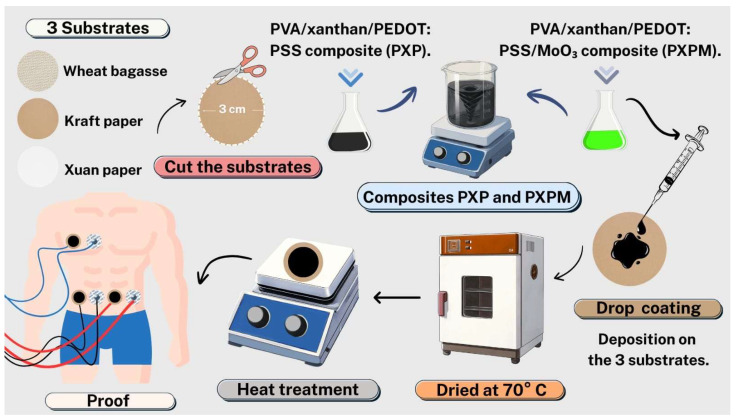
Diagram showing the manufacturing process of **PXP** and **PXPM** electrodes.

Both composites were deposited using the drop-coating technique, adding 1.5 mL of the composite to pre-cut circular substrates measuring 3 cm in diameter. Three types of cellulosic substrates were used: Xuan paper, a wheat bagasse substrate (a 100% compostable natural raw material) and Kraft paper (made from biodegradable wood pulp). Once the composites were deposited, the substrates were heat-treated to create the electrodes. First, they were left at room temperature for 12 h and then transferred to a 70 °C oven for 6 h. To complete the heat treatment, the electrodes were placed on a heat rack at 100 °C for 5 min, then on another one at 120 °C for 5 min, and finally at 140 °C for 5 min. Figure 1 shows a diagram of the complete manufacturing process.

### 2.2. Characterization of PXP and PXPM Composites and Electrodes

The optical properties of the composites and electrodes were obtained using a Unicam UV300 UV-Vis 300 spectrophotometer (Thermo Fisher Scientific Inc., Waltham, MA, USA) Fluorescence was evaluated with an FP-8550 spectrofluorometer (Jasco International, Tokyo, Japan). To evaluate electrical behavior, a programmable voltage source from Next Robotix (Mexico City, Mexico) was used along with a Keithley 4200-SCS-PK1 auto-ranging sensor (Tektronix Inc., Beaverton, OR, USA). Morphologic features were analyzed using a Hitachi SU3500 (Hitachi, Tokyo, Japan) scanning electron microscope (SEM). The thickness of the deposited films on each substrate was measured using a micrometer (Mitutoyo Corp., Kawasaki, Japan). The thickness of **PXP** and **PXPM** was found to be 30 μm on Xuan paper and 50 μm on both Kraft paper and wheat bagasse. Thickness changes are a result of differences in adhesive forces and surface tension between the composite and the substrate. Mechanical tensile tests were performed using a universal testing machine (AGS-X, Shimadzu Corp., Kyoto, Japan) with a maximum load capacity of 5 kN. To perform the test, 1 mL of **PXP** and **PXPM** composites was deposited onto substrates with dimensions of 4 cm in length and 0.942 cm in width, made of Xuan paper, wheat bagasse, and Kraft paper. The substrate thicknesses were 0.11 mm for Xuan paper, 0.5 mm for wheat bagasse and 0.33 mm for Kraft paper. Data acquisition and analysis were performed using the Trapezium X software package (version 1.5.6, Shimadzu Corp., Kyoto, Japan).

### 2.3. Evaluation of PXP and PXPM Electrodes

To characterize the performance of the developed electrodes, a single-channel ECG acquisition circuit was designed and implemented. The circuit comprised three main stages: an instrumentation amplifier with a fixed gain of 100, a fourth-order high-pass filter (0.5 Hz cutoff), and a fourth-order low-pass filter (50 Hz cutoff). The circuit was used to obtain ECG signals during two experimental phases. The first phase compared the performance of each of the manufactured electrodes against commercially available Ag/AgCl electrodes (Kendall Medi-trace 200 series, Covidien, Dublin, Ireland) in three different subjects to assess signal fidelity. The second phase aimed to demonstrate the reproducibility and robustness of the electrodes across different subjects and varying physiological states by recording ECG signals from three test subjects under three distinct conditions: at rest, while deep breathing, and during exercise. For each measurement, three electrodes were placed at the same anatomical positions: a reference electrode on the lower right side of the abdomen, a second electrode on the lower left side of the abdomen and a final electrode on the upper right side of the abdomen. The ECG signal was obtained from the voltage difference between these last two electrodes.

### 2.4. Signal Quality Index (SQI) Calculation

Due to the high susceptibility of the ECG signal to noise and artifacts, an objective assessment is required to ensure that the signal can be used for clinical purposes. To achieve this, a series of metrics known as signal quality indices (SQIs) validated in the literature [24,25] were employed to evaluate signal quality. Specifically, three SQIs were used to evaluate the signals obtained from the different electrodes: (1) the comparison of multiple beat detectors on the same signal (bSQI), (2) the evaluation of signal kurtosis (kSQI) and (3) the calculation of the spectral density of the signal within a specific physiological bandwidth (sSQI) [25].

**bSQI**: This metric is based on the principle that the detection of the QRS complex—crucial for heart rhythm analysis—varies between algorithms depending on the noise characteristics present in the signal. To implement this index, the results of two widely cited open-source QRS detectors with distinct operating principles were compared: the first relies on digital filtering and integration techniques to emphasize the energy of the QRS complex [26,27], while the second uses a length transformation after filter preprocessing [28]. The level of agreement between the detections obtained from both algorithms served as a robust indicator of overall ECG signal quality. bSQI is calculated based on the results of these two algorithms as follows [25]:

$$bSQI(k)=\frac{N_{matched}\left(k\right)}{N_{all}(k)}$$

where $N_{matched}$ is the number of beats that both algorithms detected within a time window of 150 ms, and $N_{all}$ is the number of all the beats detected by at least one of the algorithms. A good-quality ECG will have a bSQI value close to 1, indicating that all beats were detected by both algorithms.

**kSQI**: Kurtosis is a statistical metric that characterizes the shape of a signal’s probability distribution, specifically its tendency to exhibit heavy tails or outliers relative to a normal distribution. In ECG analysis, this parameter is used to quantify how closely the signal adheres to a Gaussian distribution. Clean ECG signals with sinus rhythm typically exhibit kurtosis values greater than 5 due to the prominent QRS complexes [29], making this indicator a useful tool for assessing signal quality. The kSQI is calculated as follows [24]:

$$kSQI=\frac{1}{N}\sum_{i=1}^{N}{\left[\frac{x_{i}-\bar{x}}{\sigma }\right]}^{4}$$

where $x_{i}$ refers to the i-th sample of the ECG signal of length N, $\bar{x}$ is the mean l, and σ is the standard deviation of the signal.

**sSQI:** The spectral energy of the QRS complex is predominantly concentrated in a narrow band centered around 10 Hz [30]. To assess signal quality, the power spectral density (PSD) within this specific band is compared with the PSD of the entire ECG signal. This relationship is quantified by calculating the Spectral Distribution Ratio (SDR). The SDR for an ECG segment is defined as the ratio of the power (P) of the QRS complex (5–14 Hz band) to the power of the entire ECG signal (5–50 Hz band) [25]:

$$sSQI\left(k\right)=\frac{\int_{f=5}^{f=14}P\left(k\right)df}{\int_{f=5}^{f=50}P\left(k\right)df}$$

Low sSQI values (<0.5) indicate the presence of high-frequency noise, while high values (>0.8) suggest the presence of artifacts, such as electrode motion. When the sSQI value falls within the intermediate range (0.5 ≤ sSQI ≤ 0.8), the ECG signal is of adequate quality [25]. These three SQIs were calculated for signals obtained from both commercially available electrodes and the manufactured electrodes to evaluate their comparative performance.

## 3. Results and Discussion

### 3.1. Characterization of PXP and PXPM Composites

**Figure 2:**
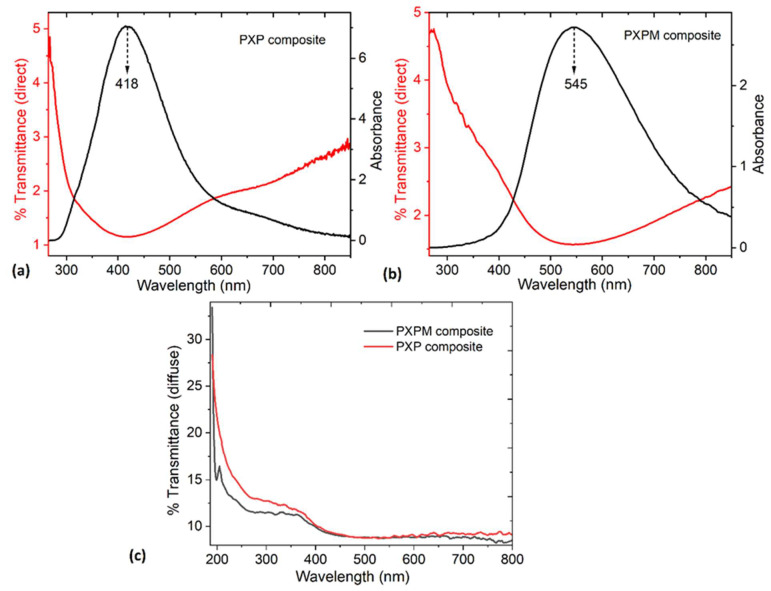
Absorbance and direct transmittance spectra of (**a**) **PXP** and (**b**) **PXPM** composites. (**c**) Diffuse transmittance spectra of **PXP** and **PXPM** composites.

The absorbance and direct transmittance spectra for the **PXP** and **PXPM** composites are shown in Figure 2a and Figure 2b, respectively. Very low absorbance is observed in both spectra. Low absorbance is desirable because it prevents the electrode from heating up due to sunlight or artificial light, which is important when in contact with the skin, where it must be kept at a stable temperature to avoid irritation or burns.

For the absorbance spectrum of the **PXP** composite, a broad band from 350 to 550 nm centered at 418 nm can be observed (violet/blue zone of the electromagnetic spectrum). This is assigned to the π-π* transition of thiophene rings in PEDOT:PSS. For the **PXPM** composite spectrum, the absorption band appears shifted to 400~750 nm and centered at 545 nm (green region); this shift is due to the MoO_3_ interaction with PEDOT:PSS [16,31]. The presence of oxide generates a broader absorption band that expands towards the near-infrared region (NIR) because of more extended electronic states, enhanced PEDOT packing, and greater PSS segregation. Due to the oxidative doping caused by MoO_3_, there is a change in the conformation of the PEDOT chains, and additional charge carriers are generated in the **PXPM** composite [32].

Figure 2a,b also show the direct transmittance of the composites, revealing very low transparency, which increases slightly towards the NIR. Direct transmittance measures only the light that passes linearly through the material; due to the roughness and heterogeneity of the **PXP** and **PXPM** composites, it is expected to obtain such low values. Therefore, and since both composites contain components that can scatter light, diffuse transmittance was also measured. Diffuse transmittance measures the light that scatters out in different directions while passing through the material. Total transmittance can be divided into two parts: direct transmittance and diffuse transmittance [33]. Although diffuse transmittance is slightly higher than direct transmittance, as shown in Figure 2c, both values remain very low, indicating strong optical absorption in both composites due to the PEDOT:PSS effect [33]. Xanthan gum and PVA enhance the dispersion on the composites, while MoO_3_ in the **PXPM** composite contributes to both absorption and scattering. If **PXP** and **PXPM** are to be used in electrode manufacturing, their low overall transparency would help improve the quality of detected signals, since the composite opacity shields the electrodes from external interference and/or optical noise. Furthermore, the low total transmittance can increase the electrodes’ resistance and stability to external factors such as sunlight, thus extending their lifespan and maintaining their functionality during prolonged monitoring procedures.

**Figure 3:**
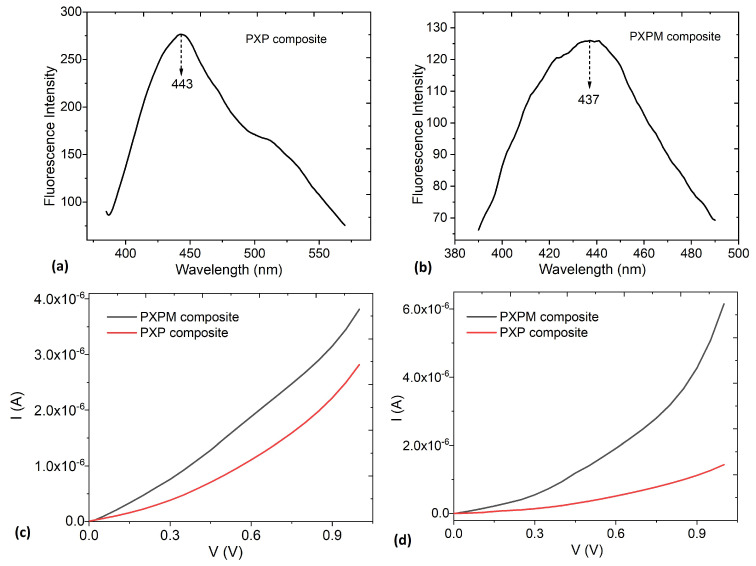
Fluorescence emission spectra of (**a**) **PXP** and (**b**) **PXPM** composites. Current–voltage graphs for **PXP** and **PXPM** composites, (**c**) new and (**d**) aged.

Figure 3a,b show the fluorescence emission spectrograms of the **PXP** and **PXPM** composites obtained at an excitation wavelength of 365 nm. It can be seen that the fluorescence emission peak of **PXP** appears at 443 nm and is sharp and strong, but that of **PXPM** is broader and centered at 437 nm [31]. In the **PXPM** composite, fluorescence intensity decreased by almost 55% (from 277 in PXP to 125 in PXPM). This reduction is attributed to the effect of MoO_3_, which generates quenching and promotes charge transfer, PEDOT:PSS doping, and non-radiative deactivation. These processes affect the excited states responsible for fluorescence and shift the maximum excitation peak towards the red region. It is important to note that the components of **PXP** and **PXPM** do not exhibit fluorescence in their pure state. Therefore, the presence of signals in the spectra may be the result of interactions between their components, the weak PSS emission, and electronic defects induced by PVA, xanthan gum, and MoO_3_. This fluorescence could be exploited in the future to monitor the degradation or aging of **PXP** and **PXPM** composite materials and to verify the formation of secondary compounds or the presence of contaminants.

To evaluate whether the new **PXP** and **PXPM** composites conduct electrical currents, the current–voltage (I-V) relationship was assessed in both materials. This is crucial, as it indicates whether the composites will function properly when interacting with ECG signals on the order of microvolts. Regarding electrical behavior, Figure 3c shows the I–V relationship, where it is evident that both **PXP** and **PXPM** exhibit almost linear and stable behavior. This behavior indicates the charge-carrying capacity of both materials, explained by the presence of PEDOT:PSS in **PXP** and, additionally, MoO_3_ in **PXPM**. This oxide has a high electron affinity, so it can extract electrons from PEDOT:PSS, leaving more holes and increasing electrical conductivity. Therefore, the I-V curve of **PXPM** shows a higher electrical current and, with this, less resistance than that of **PXP**. MoO_3_ can act as a p-type dopant in PEDOT:PSS, removing electron density from PEDOT and increasing the electric current in **PXPM** [32]. The ohmic behavior and electrical continuity of both composites suggest their potential for use as ECG electrodes, since their function is to capture bioelectrical signals from the heart and transmit them to an acquisition system without distortion.

Additionally, both composites were kept under normal temperature conditions, without any type of airtight packaging, for more than six months, and their electrical behavior was subsequently re-evaluated. This was done to determine if any type of degradation occurred in **PXP** and **PXPM**, although it is important to consider that their components have high chemical stability, and the main expected challenge is the hydrophilic nature of the PVA/xanthan gum polymer matrix. Figure 3d shows the I–V curves for the aged composites, which exhibit a less linear behavior compared to the newly manufactured materials and generate less current. This electrical behavior may be due to water loss in the **PXP** and **PXPM** structure, resulting in reduced charge transport. Table 1 presents the electrical current values at 0.5, 0.5 and 0.8 V for the composites before and after aging, as well as the percentage of loss in transported electrical current. The greatest loss occurs in **PXP**, which is an indication that the MoO_3_ in **PXPM** helps to stabilize the water molecules in the polymer network of the composite matrix. MoO_3_ improves the electrical stability of the material by acting as a stabilizing agent, promoting a higher charge carrier density, and reducing moisture-induced degradation.

Based on the previous results, electrodes were manufactured using the **PXP** and **PXPM** composites, and their morphology on the different substrates was evaluated with an SEM. The surface morphology of these electrodes determines the quality and stability of the acquired ECG signals. In this work, the use of different substrates directly influences the growth and morphology of the composite deposited on them. In particular, the presence of defects and irregularities in the composite affects the electrical resistance between the skin and the electrode. The lack of a homogeneous distribution of **PXP** and **PXPM** composites negatively influences electrical conduction and signal transmission.

**Figure 4:**
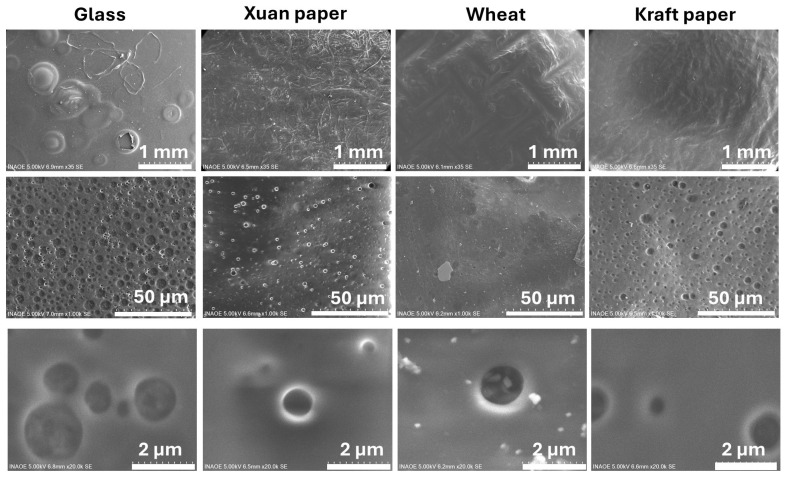
SEM micrographs comparing the surface morphology of four **PXP** electrodes. The columns show the glass, Xuan, wheat bagasse, and Kraft substrates. The rows present the surfaces at different magnifications, with reference scale bars of 1 mm, 50 µm, and 2 µm (from top to bottom).

Figure 4 shows a comparative analysis of the surface morphology of four different substrates after being coated with the **PXP** composite: glass, Xuan paper, wheat bagasse, and Kraft paper. The figure is organized as a matrix where each column represents a different substrate and each row corresponds to an increasing level of magnification (from top to bottom: 1 mm, 50 µm, and 2 µm scale bars). Column 1 shows the **PXP** composite on a glass substrate as a reference. At the 1 mm scale, the surface appears relatively smooth but with irregularities. Upon increasing the magnification to 50 µm, a highly porous surface is revealed, featuring a high density of circular domains with varying diameters, giving it a foam-like appearance. At 2 µm, these domains are observed in detail. They are well-defined, spherical, or globular cavities embedded in the composite matrix, a morphology consistent with the known phase-separation behavior of this composite film on an inert surface. During the deposition process, pore formation is mainly attributed to solvent evaporation and to phase-separation phenomena within the PEDOT:PSS-based composite. The interaction between PEDOT:PSS, glycerol, PVA and xanthan gum promotes microphase segregation during drying, leading to the formation of spherical cavities within the film matrix.

Column 2 shows the film on the surface of Xuan paper. At the 1 mm scale, the film is shown to coat the typical fibrous texture of the material. The composite is appended to the Xuan paper fibers; the wrapping of the fibers with the composite was observed, indicating that an appropriate drop-casting method was used to enable the fabrication of the **PXP** electrode with the multiscale structure of Xuan paper [20]. As the magnification increases to 50 µm, some irregular pores become visible. The magnifications at 2 µm show these pores in detail: they appear as simple circular depressions within the film, indicating that the substrate’s texture alters the morphology. However, this inherent microporous structure of the Xuan paper facilitates the penetration of the composite into the pores during the drop-coating process, ensuring a larger contact surface between pores among the micron-scale Xuan fibers [20,21].

Column 3 displays the composite on the wheat bagasse sample. At the 1 mm scale, the surface appears largely smooth, following the grooves of the underlying material. At 50 µm, the composite shows signs of poor adhesion, appearing to flake or form large blisters. Finally, at the highest magnification (2 µm), the surface is relatively uniform and grainy, lacking the distinct porous domains seen on the other substrates. Column 4 shows the film on the Kraft paper sample. At the 1 mm scale, the surface exhibits folds and wrinkles, characteristic of the paper. A significant number of pores are visible at 50 µm. However, the highest magnification (2 µm) confirms that the **PXP** composite is largely continuous and homogeneous, suppressing the well-defined pore formation seen on the glass. Wheat bagasse and Kraft paper also have a porous morphology, which causes the composite to acquire this morphology; however, porosity also guarantees the adequate introduction of **PXP** into these two substrates, as it does with Xuan paper.

In conclusion, the analysis reveals that the microstructure is dictated by the interaction between the **PXP** composite and the substrate. The composite’s intrinsic tendency to form pores is evident across the samples, but the varying textures and properties of the Xuan, wheat, and Kraft substrates significantly alter this behavior compared to what is observed on the inert glass. This results in distinct microstructures on each surface but leads to pores with a similar nature. It is also important to consider that, in all cases, complete coating of the substrates by the **PXP** composite was carried out. On the other hand, and according to Min-Hsuan Lee et al. [22], the introduction of biodegradable materials such as glycerol, PVA and xanthan gum within the PEDOT:PSS matrix can generate interconnections that induce charge transfer between the PEDOT chains, thus improving the conductivity of the composite [22,34]. This can be verified by testing the performance of the electrodes when connected to an ECG acquisition circuit. Finally, it is important to mention that the greatest number of pores is obtained in the composite deposited on glass because this substrate is not absorbent like Xuan paper, wheat bagasse and Kraft paper.

**Figure 5:**
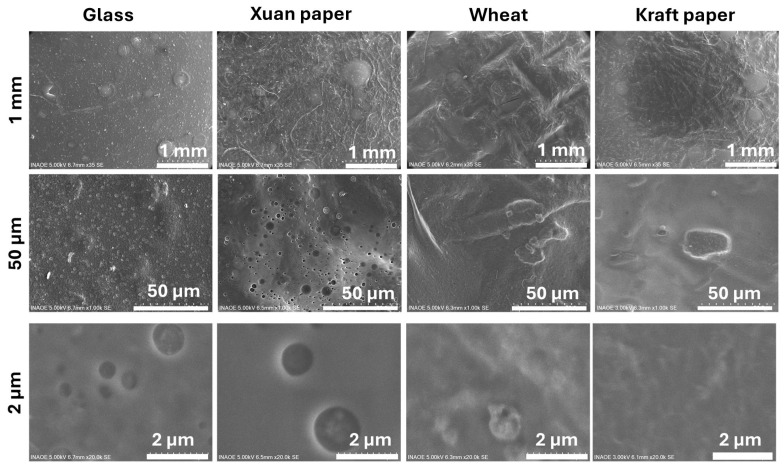
Comparative analysis by SEM of the morphology of the **PXPM** composite, deposited on four different substrates (columns). The rows show the surface at progressive magnifications, with reference scale bars of 1 mm, 50 µm, and 2 µm.

Figure 5 shows a comparative analysis of the surface morphology of four different substrates after being coated with **PXPM** composite with MoO_3_: glass, Xuan paper, wheat bagasse, and Kraft paper. The figure is organized as a matrix where each column represents a different substrate and each row corresponds to an increasing level of magnification (from top to bottom: 1 mm, 50 µm, and 2 µm scales). Column 1 shows the composite on a glass substrate as a reference. The **PXPM** composite’s morphology is very similar to that of the sample without MoO_3_: At 50 µm, a highly porous surface is revealed, featuring a high density of circular domains. At 2 µm, these domains are observed in detail as well-defined, spherical cavities, a result of the composite’s intrinsic phase-separation behavior and the low absorption of glass, which is not significantly altered by MoO_3_ on this inert surface.

Column 2 shows the composite on the surface of Xuan paper. The MoO_3_ additive also has a minimal impact here. The **PXPM** composite coats the material’s fibrous texture, and at high magnifications, the pores appear as simple circular depressions within the composite, indicating that the substrate’s texture still dictates the final morphology. Column 3 displays the composite on the wheat bagasse sample, where the effect of MoO_3_ is significant. While some irregularities are visible at 50 µm, at the highest magnification (2 µm), the film is remarkably smooth, continuous, and homogeneous, effectively suppressing the porous domain formation seen on the glass substrate. Finally, column 4 shows the film on the Kraft paper sample. Like the wheat bagasse sample, the highest magnification (2 µm) confirms that the film on Kraft paper is also very smooth and continuous, confirming that the MoO_3_ additive promotes a highly uniform coating and inhibits phase separation on this substrate as well.

In conclusion, the analysis reveals that the MoO_3_ additive has a significant and substrate-dependent effect on the film’s morphology. While it has little impact on the glass and Xuan substrates, it acts as a powerful planarization and adhesion agent on the wheat and Kraft substrates, promoting a smooth, uniform **PXPM** composite. The electrodes deposited on these two substrates are expected to be the ones that present the best acquisition of ECG signals.

The EDS data presented in Table 2 and Table 3 provides a quantitative analysis of the **PXP** and **PXPM** composites’ composition, respectively, with Table 2 establishing the baseline elemental ratio for the **PXP** composite without any additive. Table 3 confirms the presence of Mo and reveals its starkly different concentrations across the substrates, which directly correlates with the morphologies seen in Figure 4. Critically, the substrates that resulted in the smoothest, most uniform films (wheat and Kraft) show the lowest detected amounts of Mo (2.38% and 3.80% by mass, respectively). Conversely, Xuan paper, which retained a porous structure, shows the highest Mo concentration by a large margin (20.43%). These results suggest that MoO_3_ acts as an interfacial modifier that improves the interaction between the composite film and the substrate, facilitating the formation of more uniform coatings and reducing surface agglomeration of the additive; when successful, it forms a thin, buried layer that promotes a high-quality topcoat, leading to a low detection signal. A high signal, in contrast, indicates poor integration and agglomeration of the MoO_3_ additive on the surface.

The observed morphological differences among the substrates suggest that the interaction between the composite film and the underlying material plays a critical role in determining the microstructure of the deposited layer. Variations in pore formation, surface continuity, and film homogeneity may directly influence the electrical pathways within the composite. In particular, smoother and more continuous coatings are expected to improve the electrical continuity of the PEDOT:PSS-based film, reducing potential charge transport discontinuities. Consequently, these microstructural characteristics are likely to contribute to the stability and reliability of the recorded ECG signals obtained with the fabricated electrodes.

**Figure 6:**
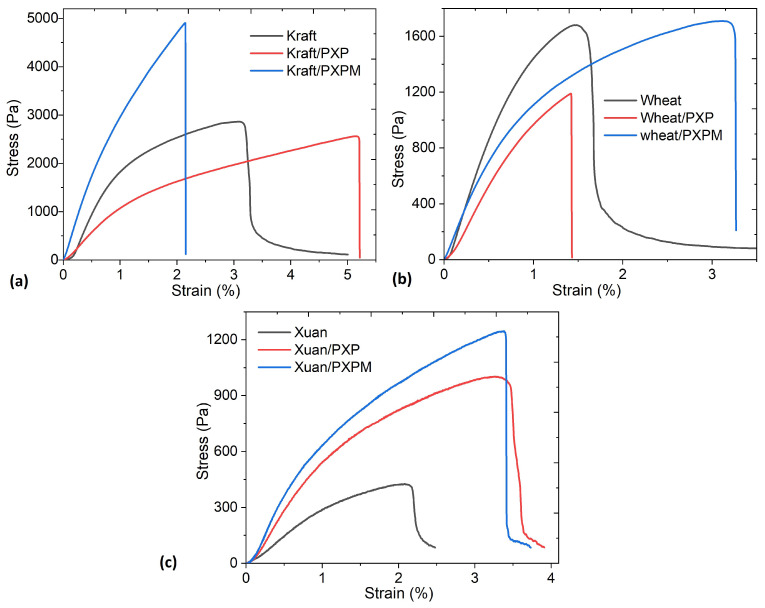
Stress–strain curve (σ-ε) of the electrodes on (**a**) Kraft paper, (**b**) wheat bagasse and (**c**) Xuan paper.

The composites must be flexible, as they must adapt to the skin surface. To determine the mechanical strength of the electrodes manufactured on the different substrates, stress–strain tests were performed for each type of **PXP** and **PXPM** electrode. The results are shown in Figure 6 and Table 4. This analysis is particularly relevant because the electrodes are intended for biomedical and wearable applications where the material is continuously subjected to bending, stretching, and cyclic deformations. Stress and strain were calculated from the force and displacement measurements obtained during the tensile tests, following the standard definitions used in mechanics of materials. Engineering stress (σ) is the intensity of force (F) distributed uniformly per unit area (A), in this case, the cross-sectional area of the electrode (see experimental section).

$$\sigma =\frac{F}{A}$$

On the other hand, engineering strain was defined as the ratio between the change in length of the sample and its initial length.

$$\epsilon =\frac{\delta }{L}$$

where δ represents the change in length or elongation and L the initial length. Finally, the elastic modulus or Young’s modulus was estimated from the slope of the initial elastic region of the stress–strain curve.

$$E=\frac{d\sigma }{d\epsilon }$$

The shape of the stress–strain curves in Figure 6 is characteristic of the presence of PEDOT:PSS, and all of them have both an elastic zone and a plastic zone. These results are similar to those presented by Zhang, L. et al. [6] for a stretchable dry electrode fabricated with PEDOT:PSS, waterborne polyurethane (WPU) and D-sorbitol and by Chen, Y. et al. [35] for a PEDOT:PSS-based tattoo electrode for long-term ECG measurement. The differences observed in the curves are attributed to the specific substrate used, since the performance of each electrode depends on the mechanical properties of the substrate used [36]. The greatest mechanical strength is obtained with Kraft paper, followed by wheat bagasse and, finally, Xuan paper. In terms of deformation, the greatest elasticity is also obtained with Kraft paper, followed by Xuan paper and, finally, wheat bagasse. Another important aspect is that the presence of MoO_3_ increases the mechanical strength of the material, especially in terms of breaking strength. A detailed analysis of Figure 6a shows that the curves of the electrodes fabricated with Kraft paper exhibit elastic and plastic regions. The presence of molybdenum increases the electrode’s rigidity, which, while significantly increasing tensile strength, decreases the percentage of deformation compared to **PXP**. Comparing the **PXP** and **PXPM** curves with the one obtained for Kraft paper reveals that each composite material has a different effect on mechanical behavior. **PXPM** increases strength, while **PXP** increases the material’s elasticity. In the case of electrodes deposited on wheat bagasse, according to Figure 6b, the presence of MoO_3_ not only increases the tensile strength and the plastic region but also significantly increases the electrode’s deformation percentage. The graph clearly shows that **PXP** does not have a significant effect, as both its mechanical strength and deformation are lower than in the substrate without the composite. Finally, the mechanical behavior for the electrodes on Xuan paper shown in Figure 6c presents elastic and plastic regions and shows that both **PXP** and **PXPM** exert similar effects in terms of deformation, although **PXPM** has a slightly higher tensile strength. In general, the best strength-to-deformation ratio is obtained for both composites on Kraft paper. When relating the mechanical behavior of the materials to their morphology (see Figure 4 and Figure 5), it is observed that the **PXP** and **PXPM** composites exhibit the most continuous, smooth, and homogeneous morphology when deposited on Kraft paper.

The values in Table 4 are well above the requirements for materials used in flexible electrodes under normal service conditions [6,35,36,37,38]. The results obtained in terms of stress and Young’s modulus are lower than those previously reported for stretchable PEDOT:PSS conductors [39]. They are also lower than those for other types of flexible electrodes, where the composite is manufactured with xanthan gum as both a thickener and glue and is deposited on Xuan paper [23]. To our knowledge, there are no studies of similar composites based on PEDOT:PSS and xanthan gum, deposited on Kraft paper or wheat bagasse; however, the Young’s modulus and breaking strength values obtained are within the minimum required for the service conditions of ECG acquisition [6,35,36,37,38] and those reported for conductive hydrogels and polymer composites used in electrodes [40]. ECG electrodes need to be manufactured from materials with mechanical properties compatible with skin, which exhibits viscoelastic behavior and whose elastic modulus is on the order of 0.1–1 kPa under small-deformation conditions [40]. In this context, elastic modulus values between 1000 and 3300 Pa, and tensile strength values between 1000 and 4900 Pa, are sufficient to guarantee conformability to the skin and mechanical stability during electrode handling and operation. The values reported in Table 3 for Kraft paper and wheat bagasse are within the lower limit, while, apparently, electrodes on Xuan paper would not meet the required mechanical characteristics. Future work could explore increasing the strength of Xuan paper, as proposed by J. Li et al. [41], by immersing it in polyvinylamine or by adding ultra-long hydroxyapatite nanowires, as reported by L.-Y. Dong et al. [42,43]. On the other hand, the deformation range is another mechanical parameter required for ECG electrodes, since, for efficient detection, it is important that the electrode be dimensionally stable. In this case, the deformation percentages for electrodes on Kraft paper are between 2.1 and 5.2, on wheat bagasse between 1.4 and 3.2, and on Xuan paper between 3.7 and 3.9, which places them above those obtained for simple PEDOT:PSS/Xuan paper electrodes [21]. The presence of xanthan gum significantly increases the deformation resistance of the electrodes.

### 3.2. Performance Evaluation of PXP and PXPM Electrodes

**Figure 7:**
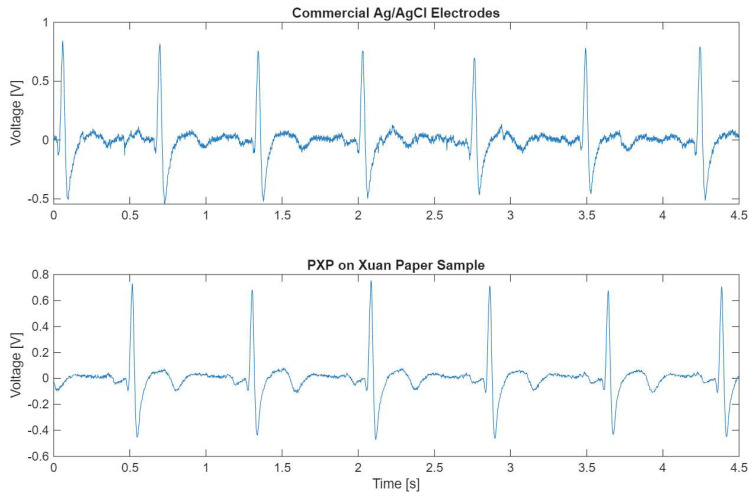
Comparison of signals obtained with commercial Ag/AgCl electrodes (**top**) and with one of the fabricated electrodes (**bottom**) connected to the same subjects, in the same anatomical positions.

ECG signals were recorded for each set of electrodes from three test subjects using consistent anatomical positioning, ensuring comparability between the signals. Figure 7 displays a comparison between signals obtained via commercial Ag/AgCl electrodes and via **PXP** on Xuan paper electrodes. The signals obtained from both sets of electrodes have the same morphology; however, the signal from the fabricated electrodes demonstrated a superior signal-to-noise ratio. This behavior was consistent across most of the signals obtained with the fabricated electrodes. Visual inspection confirms that the fabricated electrodes can record a clinically relevant signal.

Table 5 shows the results obtained for the three SQIs of each fabricated electrode compared to commercial Ag/AgCl electrodes. The primary objective of measuring these indices is to ensure that the ECG signals obtained with the fabricated electrodes can be used in a clinical setting for diagnostic purposes. A major concern when recording ECG signals is the presence of noise that can alter the morphology of the signal. Measuring the kurtosis of the signal (kSQI) has been shown to be a good indicator of a noise-free signal when the index value is greater than 5 [23,24]. All fabricated electrodes had a kSQI value greater than 5, indicating that all signals can be considered noise-free. The **PXP** sample on Kraft paper obtained the lowest kSQI value (8.785), which may indicate the presence of a small amount of noise in the ECG signal, but this is not clinically relevant. However, this result still represents a good quality signal. In contrast, both **PXP** and **PXPM** samples deposited on Xuan paper had the highest kSQI values of all the electrodes (15.114 and 13.355, respectively). This demonstrates that the performance of an electrode cannot be based only on the selection of a substrate or a composite but on the combination of both elements.

When fabricating novel electrodes, one of the main goals is to ensure a noise-free signal, for which kSQI serves as a reliable metric. Castrillon et al. [7] previously evaluated dry PEDOT:PSS electrodes using different fabric substrates, noting that their signals were consistently noisier than those of commercial Ag/AgCl electrodes. A similar trend was observed in most of the fabricated electrodes; however, the **PXP** sample on Xuan paper appears to have less noise than the commercial reference. To further validate whether these fabricated electrodes outperform commercial standards, future research should focus on testing fabricated and commercial electrodes simultaneously in different settings.

One of the primary clinical applications of ECG signals is the detection of the QRS complex, which is used to determine heart rate and heart rate variability. bSQI assesses the signal quality for identification of QRS complexes by comparing the performance of two distinct QRS detectors [26,27,28,44]. A signal with good quality is expected to have a bSQI value close to 1, indicating that the proportion of beats that are detected by both algorithms is the same. As can be observed in Table 5, all signals have a bSQI value of 1, except for the **PXPM** sample deposited on Kraft paper, which has a bSQI value of 0.993, indicating that over 99% of the QRS complexes were correctly detected. These results support those obtained from kSQI, ensuring signal quality.

The QRS complex of the ECG signal contains around 99% of the energy of the whole signal. Thus, the power distribution of the QRS complex relative to the rest of the ECG signal (sSQI) can be used as a good indicator of signal quality [23,26]. All samples exhibited sSQI values ranging between 0.557 and 0.630, which is considered normal, since a clean ECG signal is expected to yield sSQI values between 0.5 and 0.8 [24]. All **PXP** samples had a slightly higher sSQI value when compared with the commercial Ag/AgCl electrodes (0.609); however, they are still considered within the normal range for a good quality signal. In contrast, all electrodes fabricated using **PXPM** yielded sSQI values lower than those of the commercial electrodes and very similar to each other (0.559 for wheat bagasse, 0.558 for Kraft paper and 0.557 for Xuan paper). Since this value is close to 0.5, it is an indicator that half of the energy of the ECG signal is concentrated in the QRS complex, which is expected for these types of signals.

One of the main objectives of developing ECG electrodes for use with wearable devices is that the electrodes can detect a good quality signal in different situations and not just at rest, as is usually done with ECG for diagnostic purposes. To ensure that signal quality is maintained, the ECG signal was acquired from three different subjects performing three different activities: at rest, while deep breathing and while walking. The SQI values for these recordings can be found in Table 6.

When comparing the performance of the two composites, almost all **PXPM** samples exhibited higher kSQI values than their **PXP** counterparts. This can be an indication that the MoO_3_ treatment enhances electrical performance, resulting in a less noisy signal. The sole exception to this behavior occurred with the Kraft paper substrates during the walking test. Since motion artifacts are one of the biggest noise sources in ECG acquisition, it is probable that the lower kSQI values in this specific case were affected by this artifact because of physical activity.

Comparing the bSQI values in Table 6 to those in Table 5 reveals a decrease in performance, particularly for electrodes fabricated with Kraft paper. However, these electrodes outperformed dry PEDOT:PSS electrodes previously reported in the literature [45]. Specifically, the fabricated electrodes achieved a QRS detection rate of over 98% in all test conditions and with all substrates, while the referenced electrodes demonstrated a sensitivity between 63% and 98%. The difference in QRS detection proves that the presented electrodes are more reliable when obtaining an ECG signal that is clinically valid.

Across all tests, the sSQI values for the **PXPM** samples were consistently lower and closer to 0.5 compared to those for **PXP** samples. These results mirror the performance reported in Table 5, reinforcing the conclusion that the power distribution of the ECG signals acquired with the **PXPM** electrodes is closer to what is clinically expected. Consequently, **PXPM** represents a superior material choice for electrode fabrication.

SQI measurements confirmed that all fabricated electrodes can be used to obtain valid ECG signals. Furthermore, these electrodes are all made from environmentally friendly substrates and do not require conductive gel to ensure signal detection. These results are consistent with previous works [23] focusing on sustainable alternatives to traditional electrodes for use in clinical settings.

## 4. Conclusions

In this study, an easy and scalable drop-coating method was used to fabricate flexible, dry electrocardiogram (ECG) electrodes made of poly(3,4-ethylenedioxythiophene):poly(4-styrenesulfonate) (PEDOT:PSS) with polyvinyl alcohol (PVA) and xanthan gum. This composite was treated with MoO_3_, and, subsequently, both composites were characterized in terms of their optical, fluorescent, electrical, and mechanical behavior. In a subsequent stage, the two composites were deposited onto biodegradable and sustainable cellulosic substrates such as Xuan paper, Kraft paper, and wheat bagasse. The morphology of the electrodes was studied by SEM and EDS. The SEM–EDS analysis allowed for the identification of how the substrate–composite interaction governs the formation of film microstructure and the distribution of the Mo-based additive, providing insight into the mechanisms that lead to more uniform coatings and improved electrode stability. All fabricated electrodes were able to detect ECG signals successfully, with a signal quality very similar to that of commercial Ag/AgCl electrodes. Using sustainable substrates for electrode fabrication demonstrates that environmentally friendly materials do not compromise the signal-to-noise ratio or morphological accuracy required for clinical use.

## Figures and Tables

**Table 1:** Electrical current transported at different voltages in newly manufactured and 6-month-aged PXP and PXPM under normal temperature conditions and without airtight packaging.

Composite	0.3 V	0.5 V	0.8 V
I (A) **PXP**, new	3.84 × 10^−7^	8.33 × 10^−7^	1.78 × 10^−6^
I (A) **PXP**, aged	1.41 × 10^−7^	3.61 × 10^−7^	8.81 × 10^−7^
Loss in transported current (%)	63.28	56.67	50.5
I (A) **PXPM**, new	7.60 × 10^−7^	1.49 × 10^−6^	2.68 × 10^−6^
I (A) **PXPM**, aged	5.48 × 10^−7^	1.40 × 10^−6^	3.18 × 10^−6^
Loss in transported current (%)	27.9	6.04	18.7

**Table 2:** Surface elemental composition (EDS) of the PXP composite, deposited on the four substrates. The mass and atomic percentage of carbon and oxygen are quantified.

	Glass		Xuan Paper		Wheat		Kraft Paper	
	C	O	C	O	C	O	C	O
**Mass Norm (%)**	67.68	31.09	70.45	28.94	60.60	39.40	56.73	42.65
**Atom (%)**	73.92	25.49	76.20	23.50	67.20	32.80	63.73	35.97
**Abs. Error [mass%] (1 σ)**	1.86	0.98	1.92	0.92	1.67	1.22	1.58	1.30

**Table 3:** Surface elemental composition (EDS) of the **PXPM** composite with MoO_3_. The mass and atomic percentage of carbon, oxygen, and molybdenum are quantified for each substrate.

	Glass			Xuan Paper			Wheat			Kraft Paper		
	C	O	Mo	C	O	Mo	C	O	Mo	C	O	Mo
Mass Norm (%)	56.80	32.78	8.03	45.36	31.62	20.43	56.51	40.31	2.38	56.14	39.04	3.80
Atom (%)	68.00	29.46	1.20	62.22	32.56	3.51	64.63	34.62	0.34	64.99	33.93	0.55
Abs. Error [mass%] (1 σ)	2.18	1.36	0.49	2.03	1.46	1.04	1.59	1.24	0.15	1.59	1.21	0.19

**Table 4:** Mechanical properties of electrodes deposited on different substrates: Xuan paper, Kraft paper and wheat bagasse.

Sample	Young’s Modulus (Pa)	Rupture Strength (Pa)	% Strain
Wheat bagasse	1634	1680	4.9
PXP on wheat bagasse	1077	1189	1.4
PXPM on wheat bagasse	1376	1710	3.2
Kraft paper	2474	2862	5.0
PXP on Kraft paper	1157	2560	5.2
PXPM on Kraft paper	3303	4905	2.1
Xuan paper	324	421	2.4
PXP on Xuan paper	642	1001	3.9
PXPM on Xuan paper	832	1246	3.7

**Table 5:** Signal quality indices obtained for each sample of the fabricated electrodes in three test subjects, along with the Ag/AgCl commercial electrodes for comparison. Results are presented as mean ± standard deviation.

Sample	kSQI	bSQI	sSQI
Ag/AgCl electrodes	14.273 ± 3.412	1.000 ± 0.000	0.609 ± 0.061
PXP on wheat bagasse	11.223 ± 1.919	1.000 ± 0.000	0.630 ± 0.054
PXP on Kraft paper	8.785 ± 2.535	1.000 ± 0.000	0.621 ± 0.121
PXP on Xuan paper	15.114 ± 6.746	1.000 ± 0.000	0.624 ± 0.084
PXPM on wheat bagasse	11.530 ± 0.706	1.000 ± 0.000	0.559 ±0.112
PXPM on Kraft paper	12.496 ± 6.787	0.993 ± 0.012	0.558 ± 0.060
PXPM on Xuan paper	13.355 ± 7.651	1.000 ± 0.000	0.557 ± 0.039

**Table 6:** Signal quality indices obtained for each sample of the fabricated electrodes while the subjects were carrying out different activities: at rest, while deep breathing and while walking. Results are presented as mean ± standard deviation.

Substrate	Activity	PXP			PXPM		
		kSQI	bSQI	sSQI	kSQI	bSQI	sSQI
**Wheat bagasse**	Rest	11.268 ± 1.977	1.000 ± 0.000	0.630 ± 0.054	11.530 ± 0.706	1.000 ± 0.000	0.559 ± 0.112
	Deep Breathing	13.372 ± 1.447	1.000 ± 0.000	0.629 ± 0.085	16.270 ± 0.961	1.000 ± 0.000	0.557 ± 0.097
	Walking	9.807 ± 4.281	1.000 ± 0.000	0.614 ± 0.115	12.961 ± 3.159	1.000 ± 0.000	0.568 ± 0.103
**Kraft paper**	Rest	8.658 ± 2.329	1.000 ± 0.000	0.623 ± 0.124	12.508 ± 6.781	0.993 ± 0.012	0.559 ± 0.062
	Deep Breathing	11.228 ± 1.471	0.995 ± 0.009	0.628 ± 0.094	15.724 ± 7.020	0.995 ± 0.008	0.546 ± 0.110
	Walking	15.233 ± 1.812	0.994 ± 0.010	0.643 ± 0.087	9.074 ± 1.078	1.000 ± 0.000	0.567 ± 0.070
**Xuan paper**	Rest	15.114 ± 6.746	1.000 ± 0.000	0.624 ± 0.084	13.355 ± 7.651	1.000 ± 0.000	0.557 ± 0.039
	Deep Breathing	9.402 ± 2.471	0.983 ± 0.030	0.585 ± 0.122	16.056 ± 8.526	1.000 ± 0.000	0.538 ± 0.085
	Walking	7.705 ± 2.471	1.000 ± 0.000	0.540 ± 0.179	15.349 ± 8.396	1.000 ± 0.000	0.528 ± 0.086

## Data Availability

The original contributions presented in this study are included in the article. Further inquiries can be directed to the corresponding author.
